# Vedolizumab Clearance as a Surrogate Marker for Remission in Inflammatory Bowel Disease Patients: Insights from Real-World Pharmacokinetics

**DOI:** 10.3390/pharmaceutics16121629

**Published:** 2024-12-23

**Authors:** Srđan Marković, Đorđe Kralj, Petar Svorcan, Tamara Knežević Ivanovski, Olga Odanović, Sanja Obradović, Ana Homšek, Marija Jovanović, Rada Savić, Katarina M. Vučićević

**Affiliations:** 1 Department of Gastroenterology and Hepatology, University Hospital Medical Center “Zvezdara”, 11120 Belgrade, Serbia; 2 Faculty of Medicine, University of Belgrade, 11000 Belgrade, Serbia; 3 Department of Laboratory Diagnostics, University Hospital Medical Center “Zvezdara”, 11120 Belgrade, Serbia; 4 Department of Pharmacokinetics and Clinical Pharmacy, Faculty of Pharmacy, University of Belgrade, 11000 Belgrade, Serbia; 5 Department of Bioengineering and Therapeutic Sciences, University of California, San Francisco, CA 94143, USA

**Keywords:** IBD, monoclonal antibody, NONMEM, pharmacokinetics, therapeutic drug monitoring, clearance

## Abstract

**Background/Objectives:** Vedolizumab (VDZ) is approved in the treatment of patients with moderate to severe ulcerative colitis (UC) or Crohn’s disease (CD). VDZ exhibits considerable variability in its pharmacokinetic (PK) profile, and its exposure-response relationship is not yet fully understood. The aim was to investigate the variability in VDZ trough levels and PK parameters, to assess the relationship between VDZ PK and biochemical response, as well as clinical and endoscopic outcomes. **Methods**: We included 61 UC and 45 CD patients. Patients’ data and trough VDZ concentrations were retrospectively obtained. Population PK analysis was performed using non-linear mixed-effects modelling with NONMEM (version 7.5). Graphs and statistical analyses were performed using R (version 4.1.3). **Results**: In total, 116 trough VDZ concentrations from 106 patients were described by a two-compartment model. For a typical patient, clearance (CL) was estimated at 0.159 L/day, while in patients previously treated with anti-TNFα agents, VDZ CL increased by 26.4% on average. In univariate binary logistic regression, VDZ trough concentration was not statistically significant predictor of remission, whereas CL was. Moreover, combined CL and faecal calprotectin (FCP) were a statistically significant predictors of remission. The hazard ratio (HR) for CL above 0.1886 L/day was 0.35 (*p* = 0.05) and for FCP below 250 µg/g was 2.66 (*p* = 0.02) in a time-to-event analysis. **Conclusions**: Our population PK model incorporates the effect of prior anti-TNFα agents on CL, suggesting its association with more severe forms of IBD. VDZ CL emerged as a more robust and clinically relevant predictor of remission in IBD patients than trough concentration.

## 1. Introduction

Inflammatory bowel disease (IBD), which includes ulcerative colitis (UC) and Crohn’s disease (CD), considerably impairs patient quality of life due to persistent gastrointestinal inflammation. Vedolizumab (VDZ), a second-line monoclonal antibody targeting the α4β7 integrin, is approved for both the induction treatment and the maintenance of remission in patients with moderate to severe IBD. Its gut-specific mechanism of action reduces systemic immunosuppression and associated adverse effects [1,2].

VDZ exhibits considerable variability in its pharmacokinetic (PK) profile among IBD patients. At trough concentrations above 10 µg/mL, typically achieved after the repeated dosing of 300 mg every 8 weeks, VDZ demonstrates linear, non-specific elimination [3,4]. Its clearance (CL) varies among patients but generally centres around 0.16 L/day [1,5]. Similar to other monoclonal antibodies, VDZ has a volume of distribution (Vd) between 4.8 and 7.5 L [1,5,6]. This distribution pattern aligns with VDZ’s targeted binding within the gut, its large molecular size, which limits its ability to diffuse freely across cell membranes restricting tissue penetration, and its receptor-mediated CL. Consequently, the terminal half-life (t_1/2_) ranges from 15 to 25.5 days, supporting the VDZ dosing schedule every 8 weeks after the initial induction phase [1,5,7]. Despite the established efficacy of VDZ in treating moderate to severe IBD, considerable variability in patient responses remains, with some achieving clinical remission while others experience suboptimal outcomes. This variability is likely influenced by differences in drug pharmacokinetics, including CL and trough concentrations.

Population (Pop) PK modelling approach is the gold standard in identifying sources of variability among patients [8]. However, there is a scarcity of PopPK models of VDZ based on data from real-world studies [5,9,10,11,12,13]. It is important to highlight that these models were primarily developed using data generated during clinical trials, with the majority relying on the model of Rosario et al. [5]. Nevertheless, numerous publications have described and/or studied variations in VDZ trough concentrations using standard statistical approaches [6,7,14,15,16].

Thus far, several factors are recognised to contribute to the observed variability in VDZ PK. Serum albumin levels are inversely related to VDZ CL, possibly due to increased proteolytic activity or altered drug binding [5,9,10,11,12,13], with a reported cut-off albumin value of 3.2 g/dL [17]. Patients’ body weight above 120 kg significantly (and potentially clinically) increases VDZ CL leading to reduced drug exposure and possibly compromising clinical response. Similarly, the presence of neutralising anti-drug antibodies (ADAs) increases CL, but this effect usually was not considered clinically relevant [5,9,12,17]. The concomitant use of immunosuppressive therapies (such as methotrexate, azathioprine, mercaptopurine or aminosalicylates) or prior use of tumour necrosis factor-α (TNF-α) antagonist has shown contradictory results regarding its impact on VDZ CL; however, if statistically significant, it is less likely to be clinically relevant [2,17].

The relationship between VDZ trough concentration and levels of inflammatory biomarkers, clinical response(s) or remission status has been the focus of clinical trials and numerous real-world cohorts [14,15,16,18,19,20,21,22,23,24,25,26,27,28]. The majority of studies have demonstrated week-specific VDZ exposure-response relationships. These relationships include different variables among studies, reflecting the diverse methodologies and criteria used in the research. In studies where this relationship is evident, higher serum concentrations of VDZ correlate with stable C-reactive protein (CRP), haemoglobin (Hgb), erythrocytes sedimentation rate (ESR), faecal calprotectin (FCP), greater likelihood of achieving better clinical response and reaching sustained clinical remission both in CD and UC patients. These targets of trough VDZ levels greatly vary among studies. Notably, the GEMINI 1 randomised controlled trial indicated that VDZ concentration above 37.1 µg/mL at week 6 (associated with lower CL values) was linked to clinical remission at weeks 14 and 52 [19]. Similarly, a post-hoc analysis of data showed that mucosal healing rates were higher with VDZ concentrations exceeding 35.7 µg/mL at week 6 [29]. In another study by Dreesen et al., VDZ troughs > 30 µg/mL at week 2, >24 µg/mL at week 6, and >14 µg/mL during maintenance were associated with improved outcomes, including both endoscopic and clinical remission [14]. In contrast, the recent ENTERPRET study did not demonstrate a clear benefit of higher VDZ concentrations on clinical outcomes in UC patients [30]. Hence, the inconsistency across reported findings highlights the variability in results between studies, suggesting that the relationship is not yet fully understood. This underscores the need for further research in this area.

Knowledge of the efficacious target VDZ exposure, and understanding the sources of PK variability would enable clinicians to more accurately anticipate which patients will benefit most from VDZ therapy. This insight would allow for dosing regimen adjustments to improve clinical response and would help in identifying patients who may need to be switched to alternative treatments [14]. Reactive TDM of infliximab has become a critical component in the management of patients with IBD. Moreover, there is increasing evidence supporting the value of proactive TDM, which involves monitoring infliximab levels and adjusting dosing regimens before clinical symptoms arise, to optimise treatment outcomes [31,32]. Consequently, VDZ TDM is increasingly being performed in many clinical settings. The rationale for VDZ TDM includes managing the substantial interpatient variability in drug exposure, and together with clinical parameters, allowing healthcare providers to tailor VDZ dosing regimen more precisely. However, there is no universally accepted TDM protocol for VDZ, as no clear treatment time-dependent and exposure-response specific targets are being established [1,14,33].

Consequently, there is a need for further research to fully elucidate the VDZ PK variability and exposure-response dynamics of VDZ in IBD patients. Addressing these gaps underscores the need for personalised treatment approaches to optimise therapeutic outcomes and improve the clinical decision-making process.

Our research goal was to investigate the variability in VDZ trough levels and PK parameters, utilising a PopPK modelling approach on sparse TDM data. Moreover, we aimed to assess the relationship between VDZ PK and biochemical inflammatory parameters, as well as the clinical and endoscopic remission of patients with IBD in real-world clinical settings.

## 2. Materials and Methods

### 2.1. Study Design

A monocentre, retrospective, real-world study was conducted between October 2021 and August 2022, in the University Medical Center “Zvezdara”, Belgrade, Republic of Serbia, the centre of excellence for IBD in the country. The research protocol received approval from the institutional Ethics Committee (No. IRB00009457, on 5 October 2022). The study included data obtained from the medical records of all adult patients (aged over 18 years) diagnosed with either CD or UC, who were treated with VDZ therapy and who underwent serum sampling for TDM during the induction or maintenance phase. Exclusion criteria were patients’ age below 18 years, incomplete dosing and TDM data information in the medical records.

### 2.2. Treatment and Therapeutic Drug Monitoring of Vedolizumab

VDZ (Entyvio^®^, 300 mg vial solution for infusion, Takeda Italia S.P.S) was administered as 30-min intravenous infusion at a standard dose of 300 mg during the induction phase at weeks 0, 2 and 6. Maintenance dosing intervals varied among patients, occurring every 4, 6 or 8 weeks based on clinical judgement and patient response to treatment. Due to limited resources for TDM and the constraints imposed by the COVID-19 pandemic, TDM was performed randomly compared to the therapy initiation. However, it was always conducted before the next VDZ dose as an add-on to routine clinical follow-up of patients.

Blood samples, corresponding to trough VDZ levels, were drawn into 6 mL clot activator tubes. The blood samples were centrifuged at 4000× *g* for 10 min, and the sera were kept frozen at −20 °C until analysis. VDZ concentration was measured using commercially available R-Biopharm^®^ ELISA tests on Dynex DS2 analyser (Dynex Technologies, Chantilly, VA, USA) with measurement ranges from 1 to 50 μg/mL. EasyCal (Eurospital, Trieste, Italy) ready-to-use device was used for the stool sampling and pre-analytical processing of stool specimens, and the prepared extract was used for the measurement of faecal calprotectin (FCP) using the immunoenzymatic test Calprest NG (Eurospital, Trieste, Italy) on Dynex DS2 analyser (Dynex Technologies, Chantilly, VA, USA). Complete blood count and biochemical parameters were routinely analysed using standard methods. All analyses were performed in the biochemical laboratory of University Hospital Medical Center “Zvezdara”, Belgrade, Republic of Serbia.

### 2.3. Data Source

Alongside VDZ therapy and TDM data (date of VDZ therapy initiation, date of administered doses, date of blood sampling for TDM, measured VDZ concentration), prior and current concomitant therapy, detailed demographic (age, sex, body weight), disease characteristics (type of IBD, time of diagnosis, disease severity, phenotype of disease), laboratory, clinical monitoring and colonoscopy data were recorded. Detailed laboratory parameters at baseline of therapy initiation and on TDM sampling day were obtained, and they included the following: albumin (Alb), Hgb, erythrocyte (Er), iron (Fe), ferritin (FER), platelets (Plt), leukocytes (WBC), CRP and FCP. Disease activity was assessed using the Mayo score for UC, and the Simplified Endoscopic Score (SES) was used to assess endoscopic activity in CD. Complete blood count, biochemical parameters and clinical response (for UC: stool frequency, rectal bleeding and physician global assessment; for CD: stool frequency and abdominal pain) were monitored longitudinally at every visit when VDZ administration was scheduled. Clinical remission was defined as a partial Mayo score between 0 and 1 for UC, CD stool frequency ≤ 3 and abdominal pain ≤ 1. For the purpose of this study, we utilised serological and faecal biomarkers of inflammation, and clinical remission at the TDM time point. Gastrointestinal endoscopy was performed approximately every 6–12 months depending on the disease severity. Endoscopic remission was defined as a Mayo score of 0/1 for UC, and SES of 0–2 for CD, and we utilised endoscopic remission data at the subsequent time point following the TDM. Time-to-endoscopic remission was calculated from the TDM time point to the date of documented endoscopic remission or the last follow-up date for censored patients.

### 2.4. Population Pharmacokinetic Modelling

The analysis of VDZ trough concentrations was done through a non-linear mixed-effects modelling approach using NONMEM^®^ software (version 7.5) [34]. This methodology was selected for the analysis due to its proven ability to model sparse data while accounting for interindividual variability. This is essential for understanding the diverse responses in heterogeneous patient populations. Given the availability of sparse TDM data, the PRIOR approach was utilised [35,36]. This functionality allows the integration of parameter estimates from previous studies to generate more robust estimates in the presence of limited or highly variable data. We used the PopPK model developed by Rosario et al. [5] to inform and stabilise the estimation of structural and statistical model parameters. Due to the limited PK sampling time points, only CL and its variability were estimated, while for other parameters informative priors were used. The non-linear CL described by Rosario et al. was not included since it did not improve the model fit. Decay in concentration over time was described with a bi-exponential model. Between-patient variability was modelled exponentially assuming a log-normal distribution, ensuring positivity of clearance values. Additive, proportional and combined error models were tested to describe residual variability. Furthermore, the relationship between CL and demographic, treatment or clinical characteristics, including age, weight and disease severity, was explored using an automated stepwise covariate model (scm) building procedure. This procedure applied standard criteria based on the −2× log-likelihood change, which is presented in NONMEM as the objective function value (OFV). When comparing models, a difference in the OFV of 3.84, 7.88 and 10.83 are considered significant at the 0.05, 0.005 and 0.001 levels, respectively. In the covariate analysis, the inclusion criterion was *p* < 0.05, followed by an exclusion criterion of *p* > 0.01 [34]. No outliers were identified in the dataset. PopPK model evaluation is critical and involves assessing goodness-of-fit statistics, diagnostic plots and precision of parameter estimates. Iterative refinement of the model continued until a final model that adequately describes the data is achieved. Bootstrap resampling 1000 datasets was employed to estimate 95% confidence intervals (CI) for the model parameters [8]. Using the final PopPK model, we performed the simulation of the concentration-time profile after various dosing regimens using e-Campsis Pro (Calvagone, Chazay d’Azergues, France).

### 2.5. Statistical Analysis

Descriptive statistics were employed to summarise the demographic and clinical characteristics of the study population, as well as VDZ therapy and concentration data. Continuous variables are presented as mean ± standard deviation (SD) or median (interquartile range, IQR), depending on their distribution. Categorical variables are summarised as frequencies and percentages.

The Fisher’s exact or χ^2^ test was applied for the analysis of categorical variables. For paired data, the Student’s *t*-test and the Wilcoxon signed-rank test were utilised, while for unpaired data, the unpaired Student’s *t*-test or the Mann-Whitney U test was used.

To identify significant predictors of biochemical response, clinical and endoscopic remission, univariate and multivariate stepwise logistic regression and linear regression analyses were employed. Variables with a *p* < 0.05 in the univariate analysis were considered for inclusion in the multivariate model. The strength of association between predictor variables and clinical response or remission was expressed as odds ratios (ORs) with corresponding 95% CI. Cochran-Mantel-Haenszel (CMH) test was used to evaluate the association between two categorical variables while controlling for one or more strata, and appropriate ORs were calculated. To evaluate the diagnostic performance of variables in predicting clinical response and remission in IBD patients, receiver operating characteristic (ROC) curve analysis was conducted. Youden’s index was used to determine the optimal cut-off point that maximises the difference between true positive and false positive rates.

Finally, survival analysis was conducted to explore the time to endoscopic remission and to assess the impact of various variables on this outcome. Kaplan-Meier curves were generated to estimate the survival function, and a Cox proportional hazards regression model was employed to evaluate the association between various covariates and the time to endoscopic remission.

Graphs and statistical analyses were conducted with R programming language (version 4.1.3, R Foundation for Statistical Computing, Vienna, Austria) in RStudio (desktop version 1.4.1717).

## 3. Results

Our cohort included data from 106 IBD patients. The cohort consisted of 62 UC patients (mean age: 49.81 years, 58.1% male) and 45 CD patients (mean age: 49.71 years, 51.9% male). Patients were receiving VDZ as part of their standard clinical care, with concomitant immunomodulatory therapies in 81.1% of patients. Demographic and baseline biochemical characteristics are given in Table 1, while Table S1 describes their disease features.

**Figure 1:**
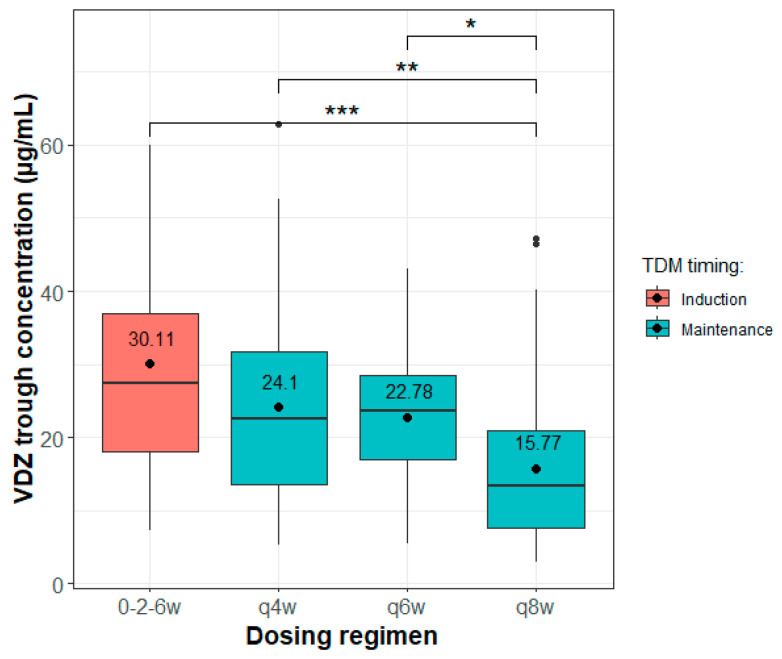
Boxplot of vedolizumab (VDZ) trough concentrations in patients receiving 300 mg as a 30-min infusion during the induction period and every 4, 6 or 8 weeks onwards. The dot represents the mean value, the central line within each box represents the median, and the edges of the box denote the interquartile range (IQR). The whiskers extend to values within 1.5 times the IQR and individual points are outliers beyond this range (* *p* ˂ 0.05, ** *p* ˂ 0.01, *** *p* ˂ 0.001).

A total of 114 VDZ trough concentration measurements (on average 1.08 samples/patient) were obtained during the follow-up period. These measurements correspond to 56 patients dosed every 4 weeks, 14 patients dosed every 6 weeks, and 36 patients dosed every 8 weeks during the maintenance phase. Due to the practical challenges posed by the COVID-19 pandemic and limited resources, TDM sampling was conducted just before the next dose but at the diverse treatment week-points during the maintenance phase, and during the induction phase. Previous studies have shown that VDZ trough levels remain stable during the maintenance phase when consistent dosing intervals are used. Accordingly, we summarised all VDZ concentrations obtained during this phase for each dosing interval. Figure 1 illustrates the boxplot of trough VDZ concentrations during the induction and maintenance period based on dosing intervals of 4, 6 or 8 weeks.

**Figure 2:**
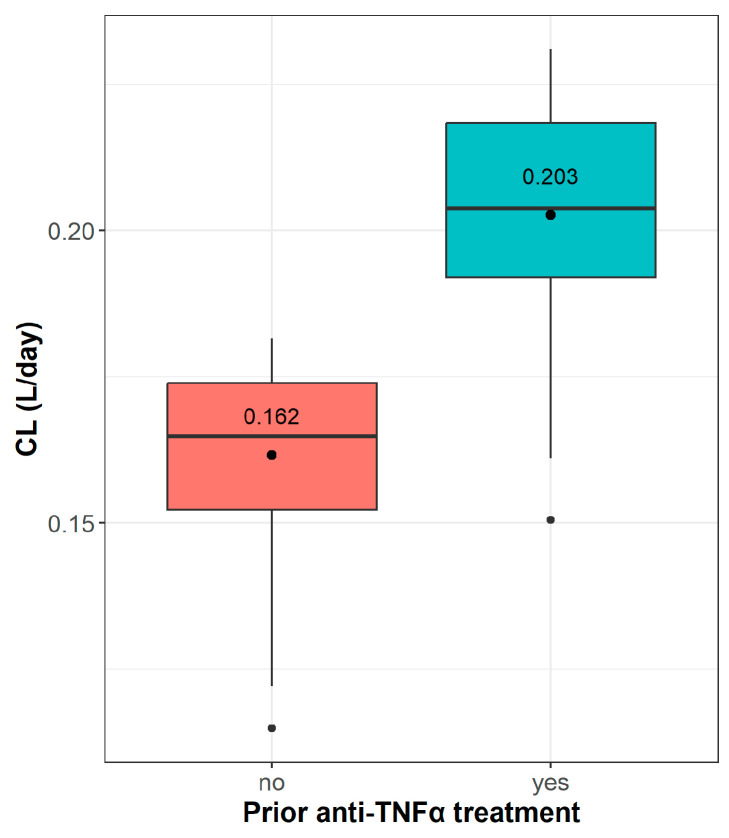
Distribution of individual vedolizumab (VDZ) clearance (CL) estimated from the final population pharmacokinetic (PopPK) model in patients with and without prior anti-TNFα therapy. The dot represents the mean value, the central line within each box represents the median CL, and the edges of the box denote the interquartile range (IQR). The whiskers extend to values within 1.5 times the IQR and individual points are outliers beyond this range.

By analysing VDZ concentration-time data and accounting for dosing history along with the exact time intervals of dosing and TDM sampling, we characterised the structural PK profile of VDZ as a two-compartment model. A proportional error model better describes residual variability. The covariates were systematically examined, resulting in no significant difference in VDZ CL between UC and CD patients. However, patients with a history of anti-TNFα therapy exhibited an average increase of 26.4% in VDZ CL, as presented in Figure 2. The relationship between typical CL and anti-TNFα therapy is given by Equation (1):

TVCL = 0.159∙(1 + 0.264∙ATNF),(1)

where ATNF equals 1 if a patient was previously treated with anti-TNFα treatment, or 0 if not. The inclusion of ATNF resulted in an OFV decrease of 13.96 units compared to the base model, corresponding to a *p*-value of 0.000187. 95% CI for the ATNF relationship parameter is 0.117–0.411, further supporting the significance of this covariate in the model.

The typical value of CL based on our final model was estimated at 0.159 L/day with an interindividual variability of 16.4%. The final parameter estimates and their bootstrap values are summarised in Table 2.

The standard goodness-of-fit plot, given in Figure S1, indicates a strong correlation between observed and model-predicted values, validating the accuracy of our model. The PopPK model was used to simulate concentration-time profiles for various VDZ dosing regimens in patients with and without prior anti-TNFα treatment, as shown in Figure S2. Based on these simulations, calculated trough concentrations and cumulative AUCs are given in Table 3.

Furthermore, we observed that serological and faecal biomarkers of inflammation stabilised and approached normal ranges at the TDM time point compared to baseline values before VDZ was introduced, at the statistically significant level *p* < 0.05 (Figure S3).

Linear regression analysis was performed to examine the relationship between biochemical parameters at the TDM time point and VDZ trough concentration during the maintenance phase or individual patient CL from the PopPK model. VDZ trough was a significant predictor of only FER levels among all the variables tested (*β* = 1.0703, *t*_(73)_ = 2.572, *p* = 0.01214), and the regression model explained 7.1% of the variance in FER. When VDZ CL was examined, the relationship was significant with CRP (*p* = 0.0302), Plt (*p* = 2.42·10^−5^), WBC (*p* = 0.000743), Alb (*p* = 0.0354) and FCP (*p* = 0.0137). Consequently, the relationship was most pronounced between VDZ CL and Plt among all tested variables. The linear regression model shows that CL is in positive association with Plt (Figure S4A), and using binary logistic regression analysis, it was found that having Plt levels greater than 350·10^9^/L is associated with significantly higher VDZ CL (Figure S4B). The area under the receiver operating curve (AUROC) value for VDZ CL in predicting the Plt categorical variable in our study was 76.9 (95% CI 62.8–91.1) with a Youden index of 0.430. However, these relationships were not statistically confirmed when analysing VDZ trough concentrations, although there was a tendency toward lower drug concentrations. The mean VDZ trough in the group of patients with Plt < 350·10^9^/L was 23.42 µg/mL versus 18.66 µg/mL in the group where Plt > 350·10^9^/L. Figure S5 shows the distribution of VDZ CL based on six categories of FCP defined by different range values.

The binary logistic regression results of the statistically significant predictors of remission are given in Table 4. VDZ trough concentration was not a statistically significant predictor of remission. Moreover, clinical remission was associated with endoscopic remission (*p* < 0.05), but with worse performance than Plt, FCP and CL, with an AUROC of 60.1%.

**Figure 3:**
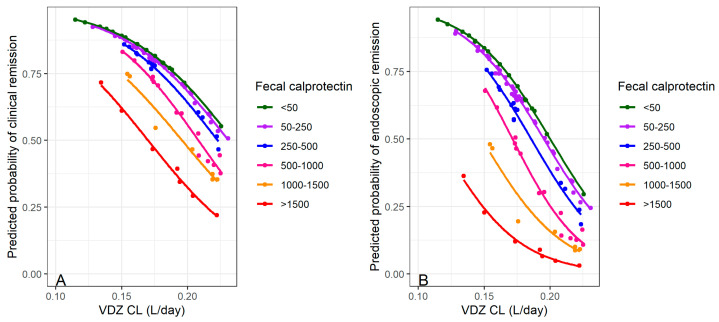
Predicted probability of (**A**) clinical and (**B**) endoscopic remission with vedolizumab (VDZ) clearance (CL) based on faecal calprotectin (FCP) values.

In our multivariate binary logistic regression, Plt was not retained in the model. We found negative coefficients for FCP and VDZ CL, indicating lower odds of clinical and endoscopic remission with increasing values of both variables, as shown in Figure 3.

The association between VDZ CL as a categorical covariate using a cut-off of 0.1886 L/day (determined by ROC analysis), and endoscopic remission was evaluated. This analysis was conducted using the Cochran-Mantel-Haenszel (CMH) test, stratified by diagnosis during the maintenance phase. The analysis indicated that the pooled odds ratio (OR) for endoscopic remission associated with CL was 0.157. Higher VDZ CL (>0.1886 L/day) was associated with a lower likelihood of achieving endoscopic remission, accounting for UC and CD. Analysis of strata-specific ORs revealed some variation, but the pooled OR indicated a consistent trend across both patient groups.

**Figure 4:**
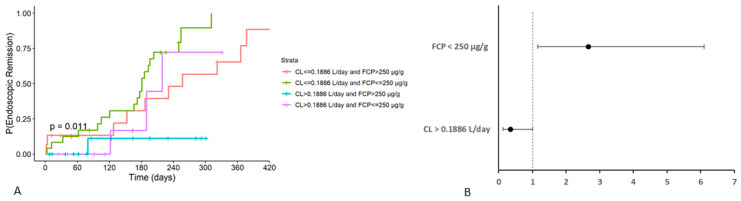
(**A**) Time to endoscopic remission based on vedolizumab (VDZ) clearance (CL) and faecal calprotectin (FCP) categories. (**B**) Forest plot illustrates the estimated effects of VDZ CL and FCP on endoscopic remission where each horizontal line represents a 95% confidence interval (CI).

Our final analysis focused on time to endoscopic remission, further supporting the previous findings. This analysis was performed in a limited number of patients (n = 64) with available data on time to remission. FCP, Plt and VDZ CL were statistically significant predictors of endoscopic remission, even though VDZ trough concentration was not. In the multivariable analysis, FCP and VDZ CL emerged as the strongest predictors of the probability of endoscopic remission (Figure 4A). Additionally, the hazard ratio (HR) for CL above the cut-off was 0.35 (95% CI: 0.12–1.0, *p* = 0.05) and for FCP below 250 µg/g, it was 2.66 (95% CI: 1.16–6.1, *p* = 0.02), as given on Figure 4B.

## 4. Discussion

The findings from this study shed light on inter-patient variability in the PK profile of VDZ, its characterisation via the PopPK model, and identifying significant factors influencing its CL in IBD patients. Furthermore, our results support that VDZ CL, rather than trough concentration, serves as a more reliable predictor of response. Given the absence of a definitive exposure-response relationship necessary to support VDZ TDM, we hypothesise that VDZ CL may act as a surrogate of response and remission in IBD patients.

The variability in VDZ PK observed in our patient cohort aligns with prior research, showing moderate to high differences in VDZ concentrations among patients. Typically, VDZ trough levels during the standard induction phase are around 27 µg/mL. In the maintenance phase, mean steady-state pre-dose levels are approximately 12 µg/mL and 36 µg/mL for dosing intervals of 8 weeks and 4 weeks, respectively, as reported in the GEMINI 1, 2 and 3 phase III studies [1,6,7,37]. When an additional dose was administered at week 10, VDZ levels were maintained around 27 µg/mL at both week 10 and week 14. The concentration then drops to expected levels with standard dosing by week 22 and remains stable thereafter [14]. High interpatient variability (above 50%) in VDZ concentrations has been consistently observed across these studies [6,7,14,37]. The trough concentrations obtained in our study (Figure 1) are consistent with these findings [2,14].

Two-compartment model best fitted observed VDZ concentrations; however, our sparse sampling design significantly limited the estimation of structural parameters other than CL. To address this limitation, we used informative priors from the previous model [5] for the remaining PK parameters and their variabilities. The estimated typical value of CL (0.159 L/day) is consistent with previous PK models, which estimated values in the range of 0.155 to 0.215 L/day [5,9,12]. Covariate analysis revealed no statistically significant difference in VDZ CL between UC and CD patients, suggesting that the disease subtype does not affect VDZ PK, as previously reported [2,5,13]. However, prior anti-TNFα therapy significantly affected CL (Figure 2 and Table 2). Specifically, patients with a history of anti-TNFα therapy exhibited an average increase of 26.4% in VDZ CL, which is similar to a previous report by Hanzel et al. (24.5% lower CL in treatment-naïve patients) [12]. Interestingly, other models describe a significant but modest effect of previous anti-TNFα therapy on CL (4–5%), judged as clinically insignificant [5,11]. However, in the exposure-response study, the probability of remission was approximately 10% higher in treatment-naïve patients [18]. We hypothesise that patients previously treated with infliximab or adalimumab may have more severe disease and inflammation, leading to gastrointestinal “leakage” that could contribute to the increased CL. This supports the potential for CL to serve as a pharmacodynamic (PD) marker for VDZ response in IBD patients, a hypothesis that should be further explored in future studies.

One key advantage of PopPK modelling is its ability to provide individual empirical Bayes estimates of PK parameters. As a result, our statistical analysis relied on patient-specific VDZ CL estimates from the final model. Further analysis revealed a positive correlation between VDZ CL and Plt levels (Figure S4A). Specifically, Plt > 350·10^9^/L was associated with higher VDZ CL (Figure S4B). A higher CL is expected to lead to lower trough concentration under the same dosing regimen. However, the relationship between trough levels and Plt was not confirmed, although a trend toward lower levels was observed. This finding may suggest that thrombocytosis reflects a higher inflammatory burden, potentially influencing VDZ elimination (Figure S4). Our results align with previous findings that lower VDZ trough levels are associated with biological markers of disease severity [14]. The role of Plts as an inflammatory marker and their association with VDZ CL is a novel observation not yet explored in existing literature [2,17]. Thrombocytosis is commonly observed in active IBD, and our study suggests that the drug’s rate of elimination may predict Plt levels. This hypothesis opens new avenues for research, particularly regarding the interplay between inflammation, thrombocytosis and VDZ trough levels or CL in IBD patients.

Previous publications have established correlations between VDZ trough levels and various responses and remissions in IBD patients [7,14,15,18,19,20,23,24,25]. The findings from our study (outlined in Table 4) contribute to the existing body of literature by indicating that VDZ CL, rather than trough concentration, is a more reliable predictor of clinical response and remission in IBD patients. Utilizing the CMH test, we demonstrated that after adjusting for diagnosis, a significant association between VDZ CL and the likelihood of achieving endoscopic remission remains both in UC and CD patients (Table 4). The cut-off point for CL as a categorical covariate (0.1886 L/day) is approximately centred within the range of previously reported typical values (0.155–0.215 L/day). In addition to CL, FCP and Plt were identified as independent predictors of endoscopic and clinical remission (Table 4), although the Plt variable was not retained in the multivariate logistic regression model. This is in line with previous findings that patients with lower FCP have a higher probability of remission [2,18]. A recent study showed that FCP < 250 μg/g at week 8 predicted endoscopic response in both UC and CD patients [38]. Additionally, other research indicated that FCP levels at weeks 16 and 52 were significantly correlated with sustained endoscopic response [39]. Our further analysis, focusing on time to endoscopic remission, supported these findings, indicating that patients with VDZ CL < 0.1886 L/day and FCP < 250 μg/g have the highest probability of achieving endoscopic remission, as presented in Figure 4.

A very important finding of our study is that changes in CL are more closely correlated with clinical and endoscopic remission than VDZ trough levels. Patients with high CL rates require higher doses to reach effective drug exposure to obtain adequate response. These results can be explained by understanding the basic key PK principles and how they apply [40]. CL is a dynamic direct measure of how efficiently the drug is being eliminated from the body, and it integrates multiple processes. In contrast, trough concentration is a static snapshot that may not fully capture the complexity of mAb elimination processes. As a result, CL offers a more comprehensive picture of the drug’s behaviour in the body. In addition, there is an intrinsic correlation between the area under the concentration-time curve (AUC), which provides a complete picture of drug exposure, and the CL of the drug. AUC is inversely proportional to CL for a given dose; thus, CL influences the overall (cumulative) drug exposure [40]. Hence, a patient with higher CL will have a lower AUC, and vice versa (Figure S2).

In addition, as previously written, VDZ CL can be influenced by various factors. The development of ADAs can increase CL and potentially reduce drug efficacy [2]. By monitoring CL, clinicians can detect the impact of ADAs and adjust treatment strategies accordingly. Trough concentration alone may not provide this insight, especially if ADAs cause rapid CL fluctuations that are not captured at a single time point. We did not measure ADA in our study, however, the rate of neutralising anti-VDZ antibodies is low at 3% in patients, so, we assume that this small percentage would not influence the study results. Therefore, by focusing on CL, not on a single concentration, we can account for these individual differences, providing a more personalised and accurate assessment of drug exposure [41]. Given that AUC represents complete drug exposure over a dosing interval, it might serve as a more comprehensive predictor of clinical response and remission compared to trough concentration alone. We hypothesise that cumulative AUC-based exposure or time above a certain threshold might better predict outcomes in IBD patients treated with VDZ than single trough concentration. Recently, preliminary results for infliximab support the AUC-response relationship suggesting AUC-guided TDM [42]. Nevertheless, some data indicate that a significant number of IBD patients gain a response over time. These are patients who did not respond to treatment at week 6 but showed a response by week 14 (approximately 10% of patients) or week 30 (around 30% of patients). This may support the importance of extended exposure to drugs over a period of time. The decision to prioritise CL over alternative exposure metrics in the analysis was driven by critical limitations in the dataset. Specifically, TDM was conducted at random intervals. Moreover, the significant variability in therapy duration and outcome timings further complicated the use of individual AUC or time above a specific target, making it challenging to account for these discrepancies. Given these challenges, simulations in a virtual population using typical dosing regimens were performed to gain insights into the underlying dynamics and better understand the situation.

However, it is important to recognise that there is a bidirectional relationship between mAb CL and disease activity. High disease activity, as seen in severe IBD, appears to increase VDZ CL, potentially due to protein loss or a “leaky gut” phenomenon, which in turn reduces drug concentrations. Conversely, elevated CL results in lower drug exposure, which may compromise clinical outcomes by failing to adequately suppress inflammation. To address this, it is crucial to first select patients where non-mechanistic failure exists, and where TDM can assist in optimising dosing to improve outcomes and achieve better disease control. Nevertheless, our ongoing prospective research aims to investigate the correlations between CL and various exposure metrics with clinical outcomes. We anticipate that this study will contribute to a more comprehensive and reliable approach to TDM of biologics.

Several limitations should be acknowledged. While providing valuable insights, the retrospective nature, the limited sample size, and the few cases in our study may limit the statistical power and restrict the generalisability of the findings. Specifically, the retrospective study design limited obtaining endoscopic results at the same time for each patient. The study was conducted at a single centre, which may introduce biases related to local clinical practices, patient demographics and treatment protocols, further limiting the external validity. Prospective multicentre studies with larger cohorts are necessary to validate these results and refine the pharmacokinetic-pharmacodynamic relationship further. Additionally, exploring the mechanisms behind the altered drug CL observed in patients with previous biological exposure could help in designing more effective treatment regimens. We acknowledge that the current frequency of 1 sample per patient during the maintenance phase may limit precision, and future studies should employ a higher sampling frequency to optimise outcome predictions. Perhaps, after the induction, the sampling before the 4th dose (week 14), every 6 months during the maintenance phase, and at flare-up of symptoms could be considered. Nevertheless, real-world clinical data are crucial for providing a more comprehensive perspective, and that is the greatest strength of this study.

In summary, while trough concentration provides useful information for TDM, VDZ CL offers a more robust and clinically relevant predictor of remission in IBD patients. By understanding CL, we can more accurately tailor dosing regimens to individual patient needs, ultimately enhancing therapeutic outcomes and achieving sustained remission. Furthermore, our ongoing prospective research aims to further investigate the potential of not only CL but also other exposure metrics as surrogates of response and remission in IBD patients. This could provide deeper insights into optimising VDZ therapy.

## 5. Conclusions

The findings of this study explore various factors of VDZ PK variability and point out a 26.4% increase in VDZ CL with prior anti-TNFα treatment. Moreover, VDZ CL emerged as a more robust and clinically relevant predictor of remission in IBD patients, potentially serving as a PD marker as it can reflect disease activity. Our results reinforce the potential role of CL as a surrogate for remission in IBD patients undergoing VDZ therapy. Future research should focus on exploring the role of VDZ CL together with identifying appropriate exposure metrics to better predict treatment response on evaluating the value of TDM and MIPD-TDM in VDZ optimisation, exploring its PK profile and exposure-response relationship in acute severe UC and fistulizing CD, and assessing its PK in paediatric IBD patients. Additionally, comparative studies of the PK profile of VDZ in comparison to other monoclonal antibodies are needed to identify potential similarities and differences that could inform overall personalised biologics therapy in IBD treatment.

## Figures and Tables

**Table 1:** Demographic and baseline biochemical characteristics of patients.

	Ulcerative Colitis (N = 62)	Crohn’s Disease (N = 44)	Total (N = 106)
Age (years)			
Mean (SD)	49.81 (17.56)	49.71 (17.44)	49.76 (17.43)
Range	21–78	21–76	21–78
Sex			
Male	36 (58.1%)	19 (43.2%)	55 (51.9%)
Female	26 (41.9%)	25 (56.8%)	51 (48.1%)
Body weight (kg)			
Mean (SD)	74.29 (11.57)	68.64 (13.74) *	71.94 (12.76)
Range	50–110	44–103	44–110
Haemoglobin (g/L)			
N-Miss	3	2	5
Mean (SD)	126.83 (23.06)	126.74 (18.85)	126.79 (21.31)
Range	75–171	68–161	68–171
Erythrocytes (·10^12^/L)			
N-Miss	5	1	6
Mean (SD)	4.50 (0.71)	4.41 (0.49)	4.46 (0.63)
Range	2.45–5.84	3.08–5.50	2.45–5.84
Iron (µmol/L)			
N-Miss	10	6 *	16
Mean (SD)	8.47 (5.84)	11.62 (6.74)	9.80 (6.39)
Range	2.0–24.8	2.8–28.2	2.0–28.2
CRP (mg/L)			
N-Miss	3	0	3
Mean (SD)	21.80 (42.97)	8.94 (13.04)	16.31 (34.10)
Range	0.1–205.0	0.1–66.2	0.1–205.0
Albumin (g/L)			
N-Miss	29	13	42
Mean (SD)	39.03 (5.75)	40.55 (6.10)	39.77 (5.92)
Range	28–49	26–52	26–52
Platelets (·10^9^/L)			
N-Miss	8	1	9
Mean (SD)	332.37 (130.65)	324.86 (118.60)	329.04 (124.87)
Range	123–797	120–701	120–797
Leukocytes (·10^9^/L)			
N-Miss	6	3	9
Mean (SD)	9.07 (3.54)	9.01 (3.50)	9.05 (3.50)
Range	2.4–18.4	2.8–16.7	2.4–18.4
Ferritin (ng/mL)			
N-Miss	15	6	21
Mean (SD)	58.83 (88.51)	45.58 (47.62)	52.91 (73.03)
Range	3–495	4–164	3–495
Faecal calprotectin (µg/g)			
N-Miss	0	1	1
Mean (SD)	608.63 (573.21)	544.19 (602.01)	582.24 (583.17)
Range	16–1801	12–2055	12–2055
Immunomodulatory drug (azathioprine, 6-mercaptopurine, methotrexate, calcineurin inhibitors)			
No	14 (22.6%)	6 (13.6%)	20 (18.9%)
Yes	48 (77.4%)	39 (86.4%)	86 (81.1%)
Prior anti-TNFα therapy			
No	39 (62.9%)	23 (52.3%)	62 (58.5%)
Yes	23 (37.1%)	21 (47.7%)	44 (41.5%)

SD-standard deviation; N-number; Miss-missing data. * *p* < 0.05 between ulcerative colitis and Crohn’s disease groups.

**Table 2:** Final vedolizumab (VDZ) population pharmacokinetic (PopPK) parameters estimate using priors and their bootstrap results.

Parameter (Units)	Estimated Value (%RSE)	Bootstrap Median (2.5–97.5th Percentile)
CL (L/day)	0.159 (4.8)	0.160 (0.143–0.177)
Vc (L)	3.19 (1)	3.19 (3.191–3.192)
Q (L/day)	0.120 (3.8)	0.120 (0.119–0.120)
Vp (L)	1.66 (1.8)	1.66 (1.65–1.66)
ATNF	0.264 (28.3)	0.263 (0.105–0.453)
IIV_CL_ (%)	16.4 (25.3)	15.9 (7.91–32.3)
IIV_Vc_ (%)	18.9 (2.8)	18.9 (18.9–18.9)
Proportional residual error	0.458 (11.1)	0.453 (0.226–0.520)

CL—clearance; Vc—volume of distribution of the central compartment; Q—intercompartmental clearance; Vp—volume of distribution of the peripheral compartment; ATNF—increase of CL in the case of prior anti-TNFα therapy; IIV—interindividual variability for corresponding parameter; RSE—relative standard error.

**Table 3:** Summary of simulation results based on the population pharmacokinetic (PopPK) model of vedolizumab (VDZ) and dosing regimens. Results are presented as medians (5–95% confidence intervals, CI).

Dosing Regimen	prior Anti-TNFα	C_trough_ (5–95% CI) [ng/mL]	AUC_0-last_ (5–95% CI) [ng·day/mL]
0–2–6w + 10w + q4w	no	36.6 (20.4–59.8)	16,534 (13,191–22,415)
	yes	25.7 (14.7–39.1)	13,851 (11,069–17,585)
0–2–6w + 14w + q6w	no	20.5 (12.4–35.9)	12,925 (9834–16,107)
	yes	13.4 (5.46–20.4)	10,236 (6877–12,993)
0–2–6w + q8w	no	11.6 (5.92–20.9)	10,860 (7856–14,551)
	yes	6.82 (2.86–13.9)	8531 (6723–12,305)
0–2–6w + 10w + q8w	no	26.4 (14.4–42.4)	11,816 (9279–16,496)
	yes	18.2 (11.4–31.8)	9879 (7380–13,386)

AUC—cumulative area under the concentration-time curve; C_trough_—trough concentration in steady-state; w—week; q4w, q6w and q8w—dosing interval of 4, 6 and 8 weeks, respectively.

**Table 4:** Summary of univariate binary logistic regression results for clinical and endoscopic remission including platelets (Plt), faecal calprotectin (FCP) and vedolizumab (VDZ) trough concentration (C_trough_) and clearance (CL) as predictors.

Predictor Variable	Remission	*p*-Value	AUROC (95% CI)	Youden’s Index	Optimal Threshold
Plt (10^9^/L)	Clinical	<0.05	57.8 (44.9–70.7)	0.249	392
	Endoscopic	<0.001	70.6 (60.6–80.6)	0.313	257.5
FCP (µg/g)	Clinical	<0.01	74.4 (64.4–84.2)	0.427	164.5
	Endoscopic	<0.001	73.0 (63.6–82.5)	0.347	190.5
VDZ C_trough_ (ng/mL)	Clinical	n.s.	54.0 (42.5–65.5)	0.155	19.84
	Endoscopic	n.s.	49.4 (38.7–60.1)	0.0887	35.71
VDZ CL (L/day)	Clinical	<0.01	67.2 (56.1–78.2)	0.287	0.1886
	Endoscopic	<0.001	72.7 (63.0–82.4)	0.442	0.1886

AUROC—area under receiver operating curve; CI—confidence interval; n.s.—not significant.

## Data Availability

The data that support the findings of this study are not openly accessible due to ethical restrictions.

## Supplementary Materials

The following supporting information can be downloaded at: https://www.mdpi.com/article/10.3390/pharmaceutics16121629/s1, Table S1: Disease phenotype and clinical characteristics of patients; Figure S1: Goodness-of-fit plots for the vedolizumab (VDZ) final population pharmacokinetic model: observed vs. (A) population (B) individual predicted trough concentration; conditional weighted residual (CWRES) vs. (C) population predicted concentration and (D) time after dose. Values are shown as points with a dashed LOESS trend line through the data, and solid is a line of identity; Figure S2: Simulated concentration-time profiles based on our final population pharmacokinetic model based on previous treatment with anti-TNFα (pink—no, blue—yes) after four dosing regimens (shadow area—95% confidence interval, CI); Figure S3: Individual biochemical parameters at baseline (0) and therapeutic drug monitoring time point (1) and calculated mean difference with p-values using paired Student’s *t*-test. (A) Hgb—haemoglobin; (B) Er—erythrocyte; (C) Fe—iron; (D) CRP—C-reactive protein; (E) Alb—albumin; (F) Plt—platelets; (G) WBC—leukocytes; (H) FER—ferritin; (I) FCP—faecal calprotectin; Figure S4: (A) Linear regression and (B) boxplot for logistic regression between vedolizumab (VDZ) clearance (CL) and thrombocytes (Plt); Figure S5: Distribution of individual vedolizumab (VDZ) clearance (CL) based on faecal calprotectin values categories. The dot represents the mean value, the central line within each box represents the median, and the edges of the box denote the interquartile range (IQR). The whiskers extend to values within 1.5 times the IQR and individual points are outliers beyond this range (* *p* ˂ 0.05).
