# Supplementary material for: Vedolizumab Clearance as a Surrogate Marker for Remission in Inflammatory Bowel Disease Patients: Insights from Real-World Pharmacokinetics
Source: Pharmaceutics. 2024 Dec 23;16(12):1629. doi: 10.3390/pharmaceutics16121629 (PMC11677246; doi:10.3390/pharmaceutics16121629)
Supplement: Supplementary file 1 [file pharmaceutics-16-01629-s001.zip › pharmaceutics-3369432-supplementary.pdf]

Supplement to Article

Vedolizumab Clearance as a Surrogate Marker for Remission in Inflammatory Bowel Disease Patients: Insights from Real-World Pharmacokinetics

Srđan Marković [1,2], Đorđe Kralj [1], Petar Svorcan[1,2], Tamara Knežević Ivanovski [1], Olga Odanović [1], Sanja Obradović [3], Ana Homšek [4], Marija Jovanović [4], Rada Savić [5] and Katarina Vučićević [4,*]

1 University Hospital Medical Center "Zvezdara", Department of Gastroenterology and Hepatology, Belgrade, Republic of Serbia
2 University of Belgrade – Faculty of Medicine, Belgrade, Republic of Serbia
3 University Hospital Medical Center "Zvezdara", Department of Laboratory Diagnostics, Belgrade, Republic of Serbia
4 Department of Pharmacokinetics and Clinical Pharmacy, University of Belgrade – Faculty of Pharmacy, Belgrade, Republic of Serbia
5 Department of Bioengineering and Therapeutic Sciences, University of California, San Francisco, CA, USA
* Correspondence: katarina.vucicevic@pharmacy.bg.ac.rs

Table S1. Disease phenotype and clinical characteristics of patients.

	Ulcerative colitis (N=62) N (%)	Crohn's disease (N=44) N (%)	Total (N=106) N (%)
**Localization of disease based on Montreal classification**			
L1: terminal ileal	-	12 (27.3)	12 (11.3)
L2: colon	-	4 (9.1)	4 (3.8)
L3: ileocolon	-	28 (63.6)	4 (3.8)
N/A	62 (100)	-	28 (26.4)
**Perianal fistula**			
Yes	-	6 (13.6)	6 (5.7)
No	-	38 (86.4)	38 (35.8)
N/A	62 (100)	-	62 (100)
**Resection**			
Yes	-	24 (54.5)	24 (22.6)
No	-	20 (45.5)	20 (18.9)
N/A	62 (100)	-	62 (100)
**Extent**			
Extensive	59 (95.2)	-	59 (55.7)
Left-sided	3 (4.8)	-	3 (2.8)
N/A	-	44 (100.0)	44 (41.5)
**Clinical remission**			
No	15 (24.2)	17 (38.6)	32 (30.2)
Yes	47 (75.8)	27 (61.4)	74 (69.8)
**Endoscopic remission**			
No	26 (41.9)	22 (50.0)	48 (45.3)
Yes	36 (58.1)	22 (50.0)	58 (54.7)

SD-standard deviation; N-number; N/A-not applicable.

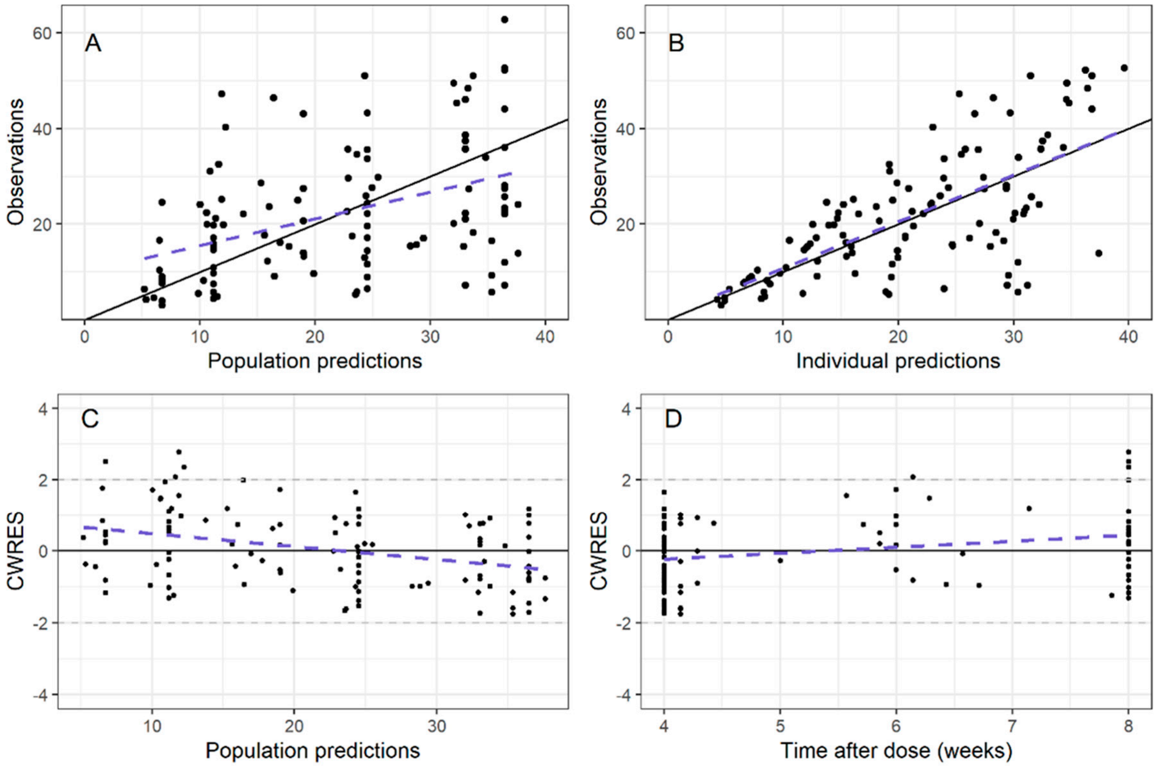

Figure S1. Goodness-of-fit plots for the vedolizumab (VDZ) final population pharmacokinetic model: observed vs. A) population B) individual predicted trough concentration; conditional weighted residual (CWRES) vs. C) population predicted concentration and D) time after dose. Values are shown as points with a dashed loess trend line through the data, and solid is a line of identity.

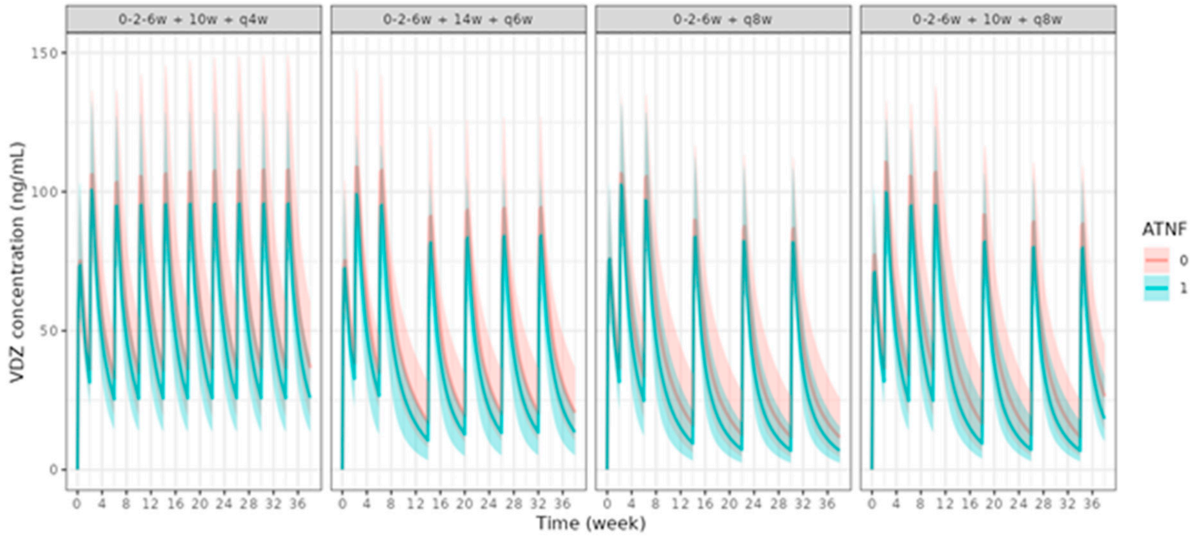

Figure S2. Simulated concentration-time profiles based on our final population pharmacokinetic model based on previous treatment with anti-TNFα treatment (pink-no, blue-yes) after four dosing regimens (shadow area - 95% confidence interval).

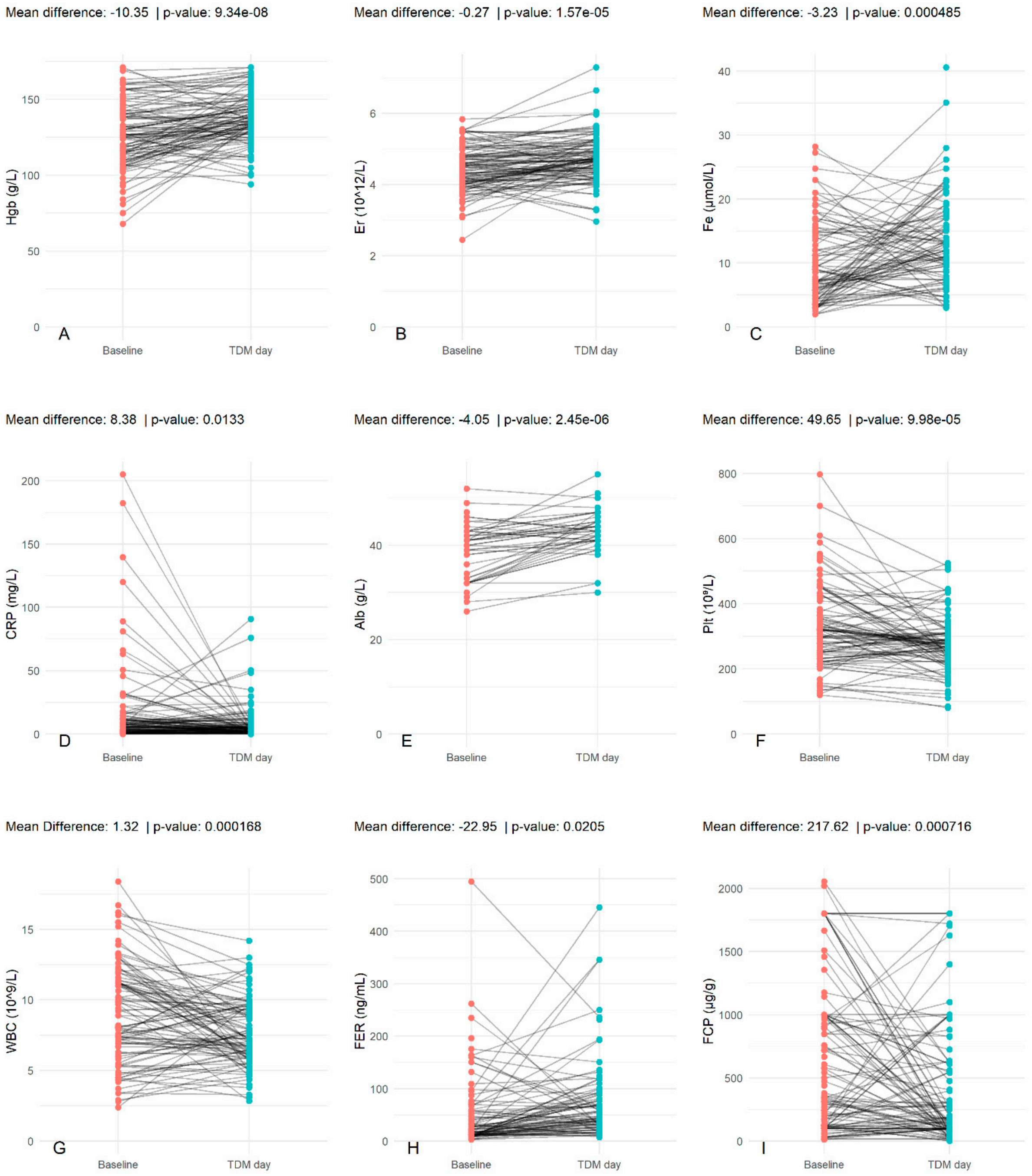

Figure S3. Individual biochemical parameters at baseline (0) and therapeutic drug monitoring time point (1) and calculated mean difference with p-values using paired Student's t-test. A) Hgb-haemoglobin; B) Er-erythrocyte; C) Fe-iron; D) CRP-C-reactive protein; E) Alb-albumin; F) Plt-platelets; G) WBC-leukocytes; H) FER-ferritin; I) FCP-faecal calprotectin.

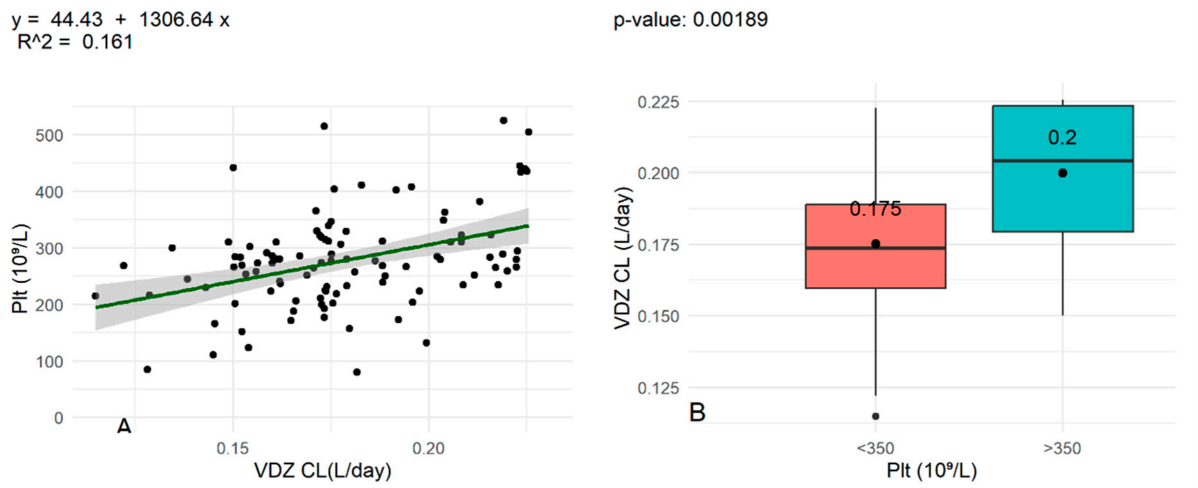

Figure S4. A) Linear regression and B) Boxplot for logistic regression between vedolizumab (VDZ) clearance (CL) and platelets (Plt).

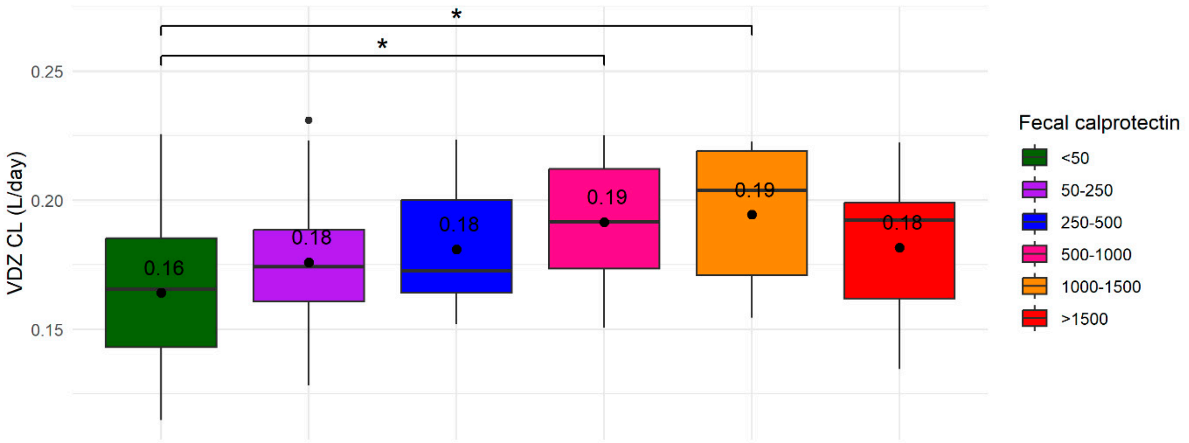

Figure S5. Distribution of individual vedolizumab (VDZ) clearance (CL) based on faecal calprotectin values categories. The dot represents mean value, central line within each box represents the median, while the edges of the box denote the interquartile range (IQR), the whiskers extend to values within 1.5 times the IQR and individual points are outliers beyond this range ($^{*}p<0.05$).
